# Interpretations of Menstrual Blood Appearance and Diagnostic Potential Among Social Media Users: Cross-Sectional Mixed Methods Social Media Listening Study

**DOI:** 10.2196/85550

**Published:** 2026-05-04

**Authors:** Ruth Niedermeier, Noemi Castelletti, Andreas Bender, Michael Hoelscher, Olena Ivanova

**Affiliations:** 1 Institute of Infectious Diseases and Tropical Medicine, LMU University Hospital, LMU Munich, Leopoldstrasse 5, Munich, 80802, Germany, 49 17661809275; 2 Fraunhofer Institute for Translational Medicine and Pharmacology, ITMP, Immunology, Infection and Pandemic Research, Munich, Germany; 3 Institute of Medical Biostatistics, Epidemiology and Informatics (IMBEI), University Medical Center, Johannes Gutenberg University, Mainz, Germany; 4 Department of Statistics, LMU Munich, Munich, Germany; 5 Munich Center for Machine Learning (MCML), LMU Munich, Munich, Germany; 6 German Centre for Infection Research (DZIF), partner site Munich, Munich, Germany; 7 Unit Global Health, Helmholtz Center Munich, German Research Center for Environmental Health (HMGU), Neuherberg, Germany; 8 SPHERE, NHMRC Centre of Research Excellence, Department of General Practice, School of Public Health and Preventive Medicine, Monash University, Melbourne, Australia

**Keywords:** menstrual blood, social media, menstrual health, diagnostic, color

## Abstract

**Background:**

Menstruation has long been framed primarily as a hygiene issue, with mainstream products and public messaging emphasizing concealment and disposal of menstrual blood (MB). This has contributed to a culture of silence in which conversations about menstrual health have been marginalized in public and clinical settings. Recent international guidance, including the World Health Organization’s call to reframe menstruation as a health issue, underscores the need for more open discourse. Simultaneously, social media has become a prominent space where menstruating individuals share experiences, seek advice, and challenge stigma. The resurgence of reusable menstrual products has increased users’ direct observation of MB, prompting questions about variations in color, texture, and smell. These developments highlight growing curiosity about MB yet reveal persistent information gaps regarding how MB is understood outside the clinical setting.

**Objective:**

This study aimed to examine how MB is represented in social media discourse and to explore individuals’ perceptions of MB’s potential use as a diagnostic tool.

**Methods:**

We conducted a cross-sectional, convergent mixed methods social listening study combining qualitative content analysis, social network analysis, sentiment analysis, and descriptive statistical analysis. Data were collected from TikTok (ByteDance), Facebook (Meta), Instagram (Meta), and Reddit using Mention and Apify. Between February 1 and 28, 2025, 6263 posts and videos were extracted using 3 strategies—group searches, hashtag searches, and social listening alerts. All data were anonymized, and demographic information was unavailable. After removing duplicates, non-English content, images, and posts without reference to blood, 349 posts were included. Coding followed a multistep deductive process in Atlas.ti. All posts were assigned with quotations, which were designated with one or more codes. Network analysis examined associations between appearance descriptors and reported health conditions. Sentiment analysis assessed perceptions of MB-based diagnostics.

**Results:**

Among the included posts (n=349), most originated from Reddit and Facebook. *Seeking help* (154/349, 44.1%) was the most common type of post. Appearance descriptions (n=243 posts) focused on color, particularly brown, bright red, pink, and black; consistency, particularly coagulation; and smell, mainly unpleasant. Network analysis linked specific colors and textures to perceived conditions, including miscarriage, endometriosis, hormonal changes, polycystic ovary syndrome (PCOS), and infections. Discussion of MB as a diagnostic tool (n=80 posts) was less frequent but included predominantly positive quotations (110/115, 95.7%), emphasizing accessibility, noninvasiveness, and home-based sampling. Concerns (19/115, 16.5%) focused on inclusivity, stigma, and bodily autonomy.

**Conclusions:**

This study demonstrates that social media serves as an important source for discussion on MB-related topics and highlights a gap between public information needs and the available scientific evidence. The findings also indicate a strong interest in MB characteristics and support further research into its diagnostic potential. To our knowledge, this is the first study to analyze social media discussions on MB characteristics and its diagnostic potential.

## Introduction

### Description of Research Problem

Menstruation has long been treated as a private hygiene issue, often framed in advertising and public discourse as something to be concealed and swiftly managed. Sanitary pads, tampons, and similar products have historically emphasized the removal and disposal of menstrual blood (MB), reinforcing a culture of silence and invisibility around menstruation. As a result, open conversations about menstrual health have long been marginalized, both in public discourse and within medical research [1].

This narrative is beginning to shift. In 2022, the World Health Organization identified 3 critical actions to advance menstrual health globally; first, to reframe menstruation as a health issue rather than a hygiene matter; second, to guarantee access to menstrual information and education; and third, to institutionalize menstrual health within governmental policies and national budgets [2]. These recommendations reflect an evolving understanding of menstruation as an important public health concern with implications for well-being, equity, and access to care.

Simultaneously, social media has become an increasingly visible space where menstruating individuals share and discuss their menstrual health in public forums. These platforms have become spaces where users share personal experiences, ask questions in moments of uncertainty, provide informal education, promote menstrual products, and challenge persistent taboos [3-7]. One significant shift contributing to this change is the resurgence of the menstrual cup, reintroduced by Mooncup in 2002 [8]. Unlike disposable products, menstrual cups allow users to observe their MB more closely, leading to greater awareness of its color, texture, and volume. This visibility often prompts questions such as “What is considered normal?” and “When should one be concerned?” [5].

Recent research on young people’s media encounters with menstruation shows that while advertising often minimizes menstrual pain and promotes a narrow, idealized portrayal of menstruation, the increasing visibility of menstruation across social and news media is simultaneously contributing to its normalization as a topic of public discussion. This ambivalence underscores social media’s dual role as both a site of stigma reproduction and a space for contesting dominant menstrual narratives [9].

Parallel to evolving societal perceptions of menstruation, medical and scientific interest in MB has increased substantially over the past decade. Historically regarded as biological waste and culturally stigmatized, menstruation is now increasingly recognized as a valuable source of biological information, reflecting both local endometrial processes and systemic physiological states [1]. Advances in analytical techniques have further enabled detailed characterization of MB, revealing its complex composition, including endometrial and immune cells, proteins, nucleic acids, lipids, extracellular vesicles, and stem or progenitor cells [10,11].

Against this background, MB is increasingly explored as a noninvasive diagnostic matrix for reproductive and systemic diseases. Studies have demonstrated the feasibility of using MB to monitor glycemic control in patients with diabetes, highlighting its potential to capture systemic metabolic biomarkers outside conventional blood sampling frameworks [12]. Similarly, passive self-collected MB has been successfully used for screening high-risk human papillomavirus, underscoring its applicability in population-level screening and self-sampling approaches [13]. More recently, technological innovation has accelerated this field, exemplified by the development of wearable in-pad diagnostic devices capable of real-time detection of disease-associated biomarkers directly from menstrual flow [14].

Beyond diagnostics, MB has also attracted attention for its regenerative and therapeutic potential. MB-derived stem cells have been extensively reviewed as a readily accessible, ethically uncontroversial cell source with promising applications in regenerative medicine, tissue engineering, and immunomodulation [15,16]. As research continues to expand, MB is increasingly recognized not only as a window into endometrial biology but also as a scalable, patient-centered biospecimen with the potential to transform approaches to disease detection, monitoring, and treatment.

Despite this growing scientific interest and increasing visibility of MB in biomedical research, little is currently known about how MB is discussed, interpreted, and made sense of by menstruating individuals outside of clinical and research settings. In this context, social media listening (SML) has emerged as a promising research method to explore public discourse around health-related topics [17]. Traditionally developed and applied by companies to monitor consumer sentiment, emerging trends, and competitor positioning across platforms, such as Facebook (Meta), Instagram (Meta), TikTok (ByteDance), Reddit, and X (formerly Twitter), SML analyses what users say and how they engage with specific topics online [18]. While rooted in commercial market research, SML has recently gained traction in the academic and medical fields as a way to access and analyze sentiments, opinions, and discourse among patients and specific social groups. For example, Carneiro et al [3] examined the discourse surrounding infertility on Instagram in Brazil; Le Busque and Mellish [19] analyzed the impact of Endometriosis Awareness Month on Instagram content; Pepin et al [20] explored the use of machine learning to analyze Reddit discussions about endometriosis; Metwally et al [21] investigated patient sentiment toward procedures like colposcopy, mammography, and Pap smears; and Sloeasen et al [22] studied how individuals with chronic ocular pain shared experiences across X, blogs, and online news platforms. These studies demonstrate the potential of SML to capture real-time, patient-centered insights that may otherwise remain undocumented in clinical settings. While scientific interest in the composition and clinical value of MB is expanding, public discourse on this topic remains largely unexplored.

### Study Aim and Objectives

In this context, this mixed methods study aims to explore how MB is discussed on social media, with two specific research questions (RQs): (1) How is the appearance of MB—such as color, texture, or smell—described and interpreted? (2) How is the potential use of MB as a diagnostic tool perceived and discussed?

## Methods

### Research Design Overview

The study used a cross-sectional, convergent mixed methods design, integrating qualitative and quantitative approaches to examine how MB is discussed on social media. This mixed methods research was chosen for studying complex health-related discourse, as it allows for both in-depth interpretation of meanings and systematic analysis of patterns and associations [23].

Qualitative and quantitative data were collected from the same social media platforms during a single extraction period and analyzed in parallel. Findings from qualitative content analysis were integrated with results from descriptive statistical analysis, social network analysis, and sentiment analysis during interpretation. The qualitative objective was to explore how menstruating individuals describe and interpret the appearance of MB, including color, texture, and smell, in social media discourse and how individuals link MB characteristics to specific menstrual health conditions. The quantitative objective was to examine patterns in these descriptions and to assess sentiment and relational associations between MB characteristics and perceived health conditions using descriptive statistics and sentiment analysis. Ultimately, we integrated the qualitative interpretations with quantitative patterns to better understand how experiential knowledge, perceived diagnostic meaning, and attitudes toward MB-based diagnostics emerge and interact in public online discourse.

SML was selected because it enables the examination of naturally occurring discussions in public online environments. Hence, potential researcher bias could be avoided. Compared with surveys or interviews, SML captures spontaneous, user-generated discourse and reveals terminology, interpretations, and concerns that may not emerge in traditional qualitative research. However, SML also has methodological constraints, including platform-specific biases, variable data accessibility, and the absence of demographic information [17].

### Data Sources and Sampling

The unit of analysis was individual social media posts rather than identifiable users. Data were collected from TikTok, Instagram, Facebook, and Reddit using Mention and Apify. These platforms were selected because they represent some of the most widely used social media globally, with Facebook reporting 3.07 billion monthly active users, Instagram 3 billion, TikTok 1.99 billion, and Reddit 765 million users [24]. Other major platforms (eg, YouTube [Google], WhatsApp [Meta], and WeChat [Tencent Holdings Limited]) were excluded due to limited data accessibility, privacy restrictions, and technical constraints, such as restricted downloading or transcription options. Posts represented the spontaneous discourse of platform users; individual-level demographic information was not available because all data were anonymized. No direct interaction with users occurred. Posts were eligible if they were publicly accessible, in English to ensure analytical consistency, contained explicit reference to MB, and were retrievable through one of the 3 extraction methods. Posts were excluded if they consisted of images or video without analyzable text, were in non-English languages, were duplicates, or did not mention the word “blood.” Visual data were excluded because consistent, valid visual analysis was not feasible in this study, and only textual descriptions of MB appearance were analyzed.

### Data Collection

Data extraction was conducted following principles of SML analysis in health research as previously described [4,19,25]. New user profiles were created on Instagram, TikTok, Reddit, and Facebook to minimize algorithmic bias. The profiles were gender-neutral and unlabeled, with the exception of the Instagram account, which required a sex designation and was therefore registered as “female.” Data were collected between February 1 and February 28, 2025. However, this time frame refers to the time of extraction, rather than to the time of publication of the posts. To improve data quality and extend the time frame in which posts were published, we applied triangulation and used 3 methods to extract content from these social media platforms [26].

Group searches: Facebook and Reddit groups were preselected by searching 4 keywords, which were selected by the researchers, as they closely align with the study focus (“Menstrual blood,” “periods,” “period blood,” and “endometriosis” [this term chosen due to the main focus of MAI study (Menstrual Blood Analysis with AI]) and screening the top 10 groups or communities per keyword. After removing duplicates and assessing relevance, 3 Facebook groups (“Girl Talk With Dr. Amber: Menstrual Problems + Solution.Period.,” “PERIOD PROBLEM,” and “Menstruation (Period) share problem”) and 1 Reddit community (“r/Periods”) were included. Using Apify’s Facebook Group Scraper and Reddit Scraper Lite, the 4 preselected groups or communities were scraped.

Hashtag searches: In total, 2 hashtags (#periodblood and #menstrualblood), which are widely used and directly refer to MB-related keywords as selected in the group search, were chosen. Apify’s Hashtag Scraper tools retrieved all posts on TikTok and Instagram associated with these hashtags at the time of extraction. Because Apify’s hashtag scrapers limit retrieval to a maximum of 100 posts per hashtag, researchers could not influence or verify the completeness of the extracted content beyond initiating the search and, therefore, could not ensure that all relevant posts associated with each hashtag were captured.

Social listening alerts: In total, 6 alerts were created to capture MB-related discussions across platforms over 2 time frames. A prospective search collected and downloaded posts from February 13 to 19, 2025; a retrospective search collected posts from February 2023 to January 2025. Alerts included combinations of menstrual-related terms (eg, “menstrual blood,” “period blood,” “clots,” and “color”). Platforms included in alerts were TikTok, Instagram, Facebook, and X (Twitter).

**Figure 1.**
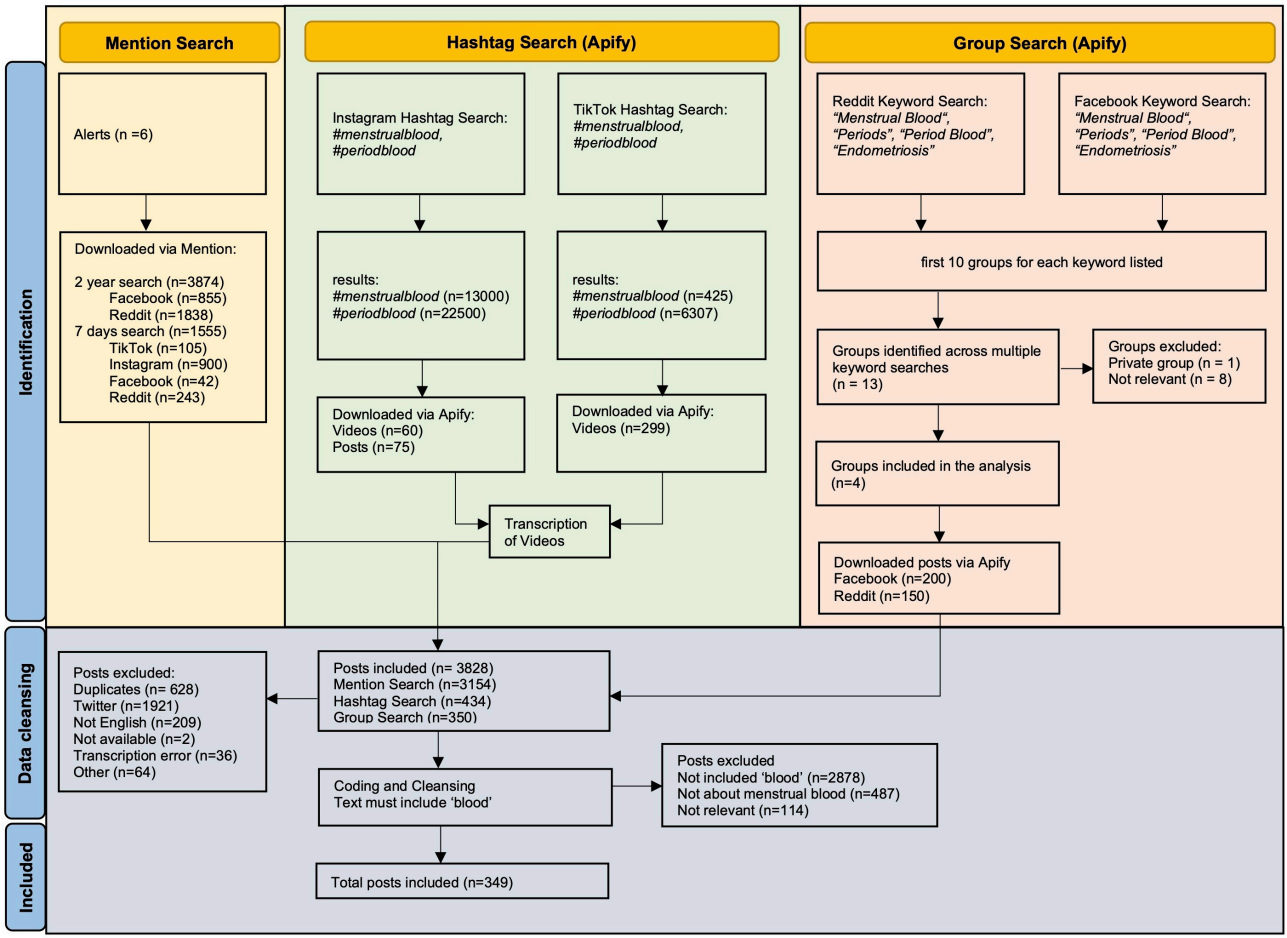
Identification and downloading process: Flowchart illustrating the data identification, extraction, and filtering process used in this mixed methods social media listening study. Data were retrieved from TikTok, Instagram, Facebook, and Reddit in a prospective search from February 13 to February 19, 2025, and a retrospective search from February 2023 to January 2025 using group searches, hashtag searches, and social listening alerts. The figure shows the steps from initial retrieval (6213 posts) to the final analytic dataset (349 posts).

In total, 6213 posts and videos were retrieved before filtering. Figure 1 illustrates the data collection and preparation workflow. A detailed description of each search method can be found in Tables S1-S5 in Multimedia Appendix 1.

### Transforming the Data

All data were anonymized before analysis. Usernames, profile information, timestamps, and identifiable details were removed. Content from X was excluded because it could be extracted exclusively through the alert search and was, therefore, lacking the quality principle of triangulation. After removing duplicates and X content, 3664 posts remained. Data were categorized by type (images, videos, and text). Images were excluded as they could not be objectively analyzed. A total of 392 videos from Instagram and TikTok were transcribed using OpenAI Whisper in Python. Posts in other languages rather than English were excluded. Using a keyword search for the term “blood,” 2890 nonrelevant posts were removed. A final relevance screening yielded 349 posts for analysis.

### Data Analysis

#### Overview

Similar to recent SML studies [4,6,27,28], we used a mixed methods analysis to examine how MB was described (RQ1), which companies working with MB were mentioned, and what benefits and concerns were discussed regarding its diagnostic use (RQ2), with categories treated as nonmutually exclusive.

#### Qualitative Analysis

##### Content Analysis

Analysis followed a multistep deductive approach. The dataset was coded using an iterative, cycling approach. First, Atlas.ti’s AI Coding tool generated an initial set of codes. The first author (RN) manually read and reviewed the entire dataset, revising, refining, and verifying all artificial intelligence (AI)–generated codes before applying them to the dataset. Additionally, new codes were applied. Moreover, 2 authors (RN and OI) discussed and refined the codes. All duplicates were removed, similar codes were merged, and a structured codebook was created.

For analytical clarity, 3 levels of analysis were distinguished: “posts” as the contextual unit of analysis, “quotations” as the coding unit, and “codes” as analytical categories. Posts represented complete social media entries and were each assigned to a single post type (eg, *explaining* and *seeking help*). Quotations referred to discrete text segments within posts that contained relevant content and could be assigned to one or more codes. Codes were applied at the quotation level to capture specific thematic elements, allowing multiple codes from different code groups to be assigned to the same quotation where applicable. As defined by Atlas.ti, groundedness describes the number of quotations coded by a specific code [29].

To address RQ1 on the appearance of MB, 4 code groups were developed: *consistency description*, *color description*, *smell description*, and *other appearance description*. For RQ2 on the diagnostic use of MB, 7 code groups were used: *benefits*, *companies*, *hopes for the future*, *concerns*, *diagnostic methods*, *research*, and *other*. Additional cross-cutting category codes included *type of post*, *sentiment*, and *reasons for MB changes*, supporting contextual analysis of content and tone.

In total, 209 individual codes were generated and are detailed in Tables S6 and S7 in Multimedia Appendix 1.

Given that all eligible posts were coded and the final stage of coding did not yield new categories beyond those already established, data saturation was considered achieved.

##### Network Analysis

To address RQ1, we performed a network analysis, a methodological approach that examined the relation (edges) between individual actors (nodes), in order to explore how specific characteristics of MB were associated with various health conditions [30,31]. Using Atlas.ti’s network analysis, we linked codes that described appearance descriptions to specific health conditions, as perceived by social media users.

### Quantitative Analysis

Statistical analysis: To examine the distribution of posts by platform, RQ, search type, and country, we performed a descriptive statistical analysis.

Sentiment Analysis: Next, we also carried out a sentiment analysis to investigate public perceptions of using MB as a diagnostic tool (RQ2) [21,22]. Sentiment analysis was conducted manually. In addition to content-related codes, each post discussing MB-based diagnostics was categorized as positive, negative, or neutral based on explicit evaluative language by the first author (RN). The last author revised all sentiment-related codes. Positive posts included statements expressing trust, enthusiasm, or implied willingness to use MB as a diagnostic tool. Negative posts expressed concerns, distrust, skepticism, or implied aversion to the use of MB for diagnostics. Neutral posts discussed the potential use of MB without evaluating the wording.

### Validity, Reliability, and Methodological Integrity

Methodological rigor was ensured through strategies appropriate to both qualitative and quantitative components of this mixed methods study, in line with Journal Article Reporting Standards for Qualitative and Quantitative Research [32].

For the qualitative analysis, an initial set of deductive codes was generated and refined through repeated engagement with the data. To further enhance reliability, all codes were discussed by 2 authors (RN and OI). Data saturation was considered achieved when no new codes or themes emerged during the final stages of analysis. The dataset was adequate to address the study aims, as it captured diverse, naturally occurring discussions of MB across multiple platforms (Reddit, Facebook, Instagram, and TikTok) and time frames. Although individual demographic information was unavailable, platform diversity and triangulated extraction strategies enhanced the breadth of perspectives represented.

For the quantitative components, reliability was supported through systematic and replicable procedures for data extraction, filtering, and categorization. Descriptive statistics, network analysis, and sentiment categorization were applied consistently across the full dataset. Network analysis followed established methodological principles to examine associations between MB characteristics and perceived health conditions, while sentiment analysis applied predefined evaluative criteria (positive, negative, and neutral) based on explicit user statements.

### Ethical Considerations

This study involved the analysis of publicly available social media content and did not include interaction with users or the collection of identifiable personal information. The study protocol was reviewed and approved by the Ethical Commission of Ludwig-Maximilians-University Munich (Project 24‐1112). Because we relied exclusively on publicly accessible posts and did not recruit participants, informed consent was not required. To protect privacy and confidentiality, all data were anonymized before analysis. Usernames, profile information, timestamps, images, and any potentially identifying material were removed during data preparation, and no attempts were made to reidentify individuals. Only deidentified textual content was retained for coding and analysis—consistent with ethical standards for social media research. As no human participants were recruited for the study, no compensation was provided. No identifiable images, profile screenshots, or user photographs were included in the manuscript or supplementary material. All figures and visualizations reflect aggregated or abstracted data. Therefore, no individual user can be identified, and no additional consent is required for publication.

## Results

### Quantitative Post Analysis

In total, 349 social media posts discussing MB were analyzed (n=349). Table 1 illustrates the distribution of search terms, sources, and geography. *Seeking help* was the most common post type (154/349, 44.1%), followed by *comment* (73/349, 20.9%), mainly on Reddit and Facebook. *Explaining* posts (47/349, 13.5%) were found across all platforms, especially Facebook and Instagram, while *advertising* (35/349, 10%) occurred mostly on Facebook. Less common were *patient reports*, *spiritual*, and *menstrual awareness*.

**Table 1.** Search tools, sources, and geography of retrieved social media posts: summary of all extraction tools (Apify scrapers and Mention alerts), platforms included (TikTok, Instagram, Facebook, and Reddit), the type of search performed, and the geographic metadata available for retrieved posts. This quantitative data reflects filtered and preprocessed content collected during the extraction period (N describes the entire set of included posts and n/N describes the proportion within a specific subgroup).

	Values, n (%)	Values, n/N (%)
Total included posts	349	—^a^
Search tool and Search terms		
Apify	27 (7.7)	27/27 (100)
GS_Facebook	7 (2)	7/27 (25.9)
GS_Reddit	7 (2)	7/27 (25.9)
HS_TikTok	6 (1.7)	6/27 (22.2)
HS_Insta_Post	4 (1.1)	4/27 (14.8)
HS_Insta_Video	3 (0.9)	3/27 (11.1)
Mention	322 (92.3)	322/322 (100)
HS_MB + Endo	192 (55)	192/322 (59.6)
HS_PB and period facts	13 (3.7)	13/322 (4)
MB and Endo	102 (29.2)	102/322 (31.7)
MB+ #	9 (2.6)	9/322 (2.8)
MB+color+diagnostics	6 (1.7)	6/322 (1.9)
Source		
Facebook	92 (26.4)	—
Instagram	24 (6.9)	—
Reddit	226 (64.8)	—
TikTok	7 (2)	—
Geographies		
Unknown	278 (79.7)	—
Known	71 (20.3)	71/278 (100)
Austria	7 (2)	7/278 (9.9)
Botswana	1 (0.3)	1/278 (1.4)
Canada	2 (0.6)	2/278 (2.8)
Cameroon	2 (0.6)	2/278 (2.8)
Egypt	1 (0.3)	1/278 (1.4)
Spain	1 (0.3)	1/278 (1.4)
Great Britain	8 (2.3)	8/278 (11.3)
India	8 (2.3)	8/278 (11.3)
Luxembourg	3 (0.9)	3/278 (4.2)
Malaysia	1 (0.3)	1/278 (1.4)
Nigeria	5 (1.4)	5/278 (7)
Netherlands	1 (0.3)	1/278 (1.4)
New Zealand	2 (0.6)	2/278 (2.8)
Philippines	1 (0.3)	1/278 (1.4)
Portugal	1 (0.3)	1/278 (1.4)
Taiwan	1 (0.3)	1/278 (1.4)
United States	25 (7.2)	25/278 (35.2)
Zimbabwe	1 (0.3)	1/278 (1.4)

a Not applicable.

Platform-specific trends showed Reddit posts (n=226) were largely focused on *seeking help* (144/226, 63.7%), while Facebook (n=92) featured more *advertising* (28/92, 30.4%) and *explaining* (21/92, 22.8%). Instagram (n=24) and TikTok (n=7) posts were primarily *explaining* (13/24, 54.2% and 6/7, 85.7%, respectively; Table S8 Multimedia Appendix 1).

Regarding the research focus, most posts addressed appearance-related aspects of MB and were thus grouped into RQ1 (243/349, 69.6%), and 80 discussed its diagnostic potential (80/349, 22.9%) and were grouped into RQ2, among those only a few were overlapping (5/349, 1.4%), whereas some could not be assigned to a specific RQ (31/349, 8.9%). RQ1 content was mainly sourced from Reddit (183/243, 75.3%), whereas RQ2 was mostly discussed on Facebook (49/80, 61.3%) and Reddit (27/80, 33.8%).

### RQ1: Qualitative and Quantitative Description of the Appearance of MB

On posts describing MB appearance (n=243), 80 codes from 4 code groups were distributed among 295 quotations: *color*, *consistency*, *smell*, and *other characteristics*.

#### Color, Consistency, Smell, and Other Descriptions

**Figure 2.**
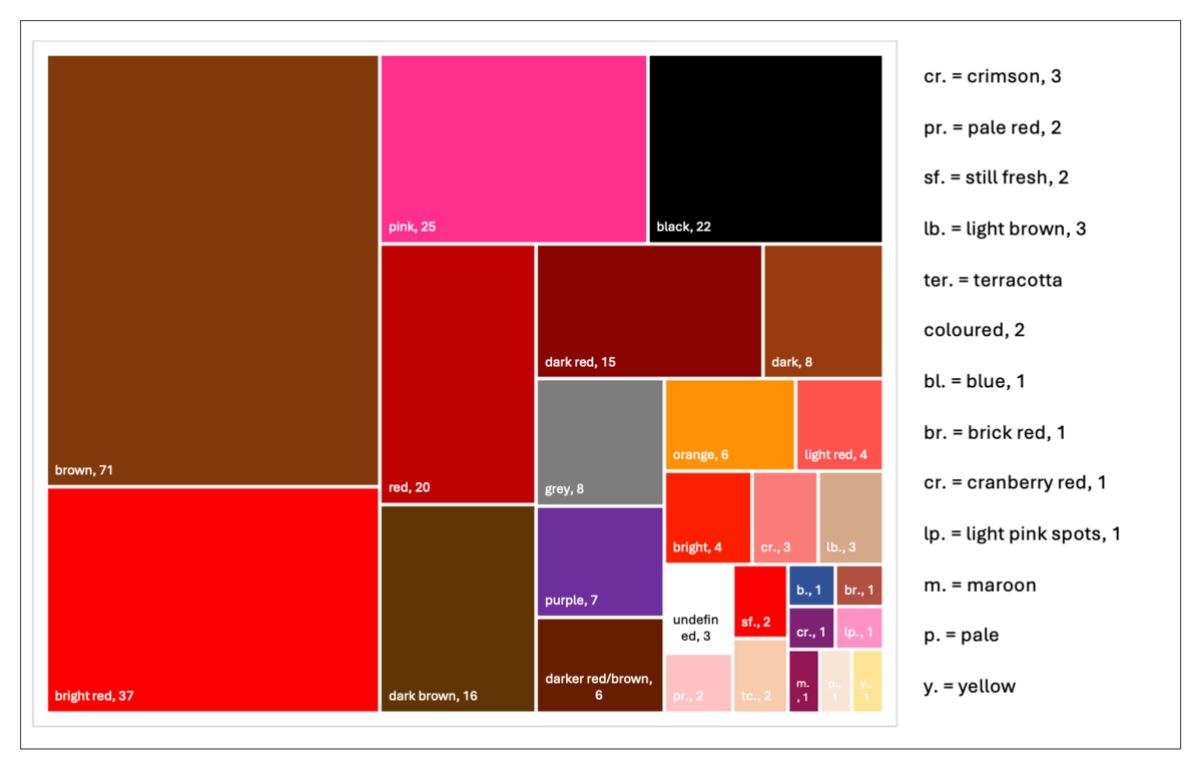
Tree map visualization of color descriptions mentioned in posts: Tree map visualization of color descriptors used by social media users to describe menstrual blood in the final dataset. The figure displays the relative frequency of specific color categories and illustrates how users characterize menstrual blood appearances. Each rectangle represents a different color, and the size is proportional to the number of posts mentioning that color. The number after the color indicates the number of mentions.

Color was the most frequently described aspect (189/295, 64.1%), with *brown* (71/189, 37.6%), *bright red* (37/189, 19.6%), *pink* (25/189, 13.2%), and *black* (22/189, 11.2%) among the most common. Other colors included *dark red, gray, purple, orange*, and unique terms like *cranberry red* and *terracotta*, as illustrated in Figure 2.

Consistency (123/295, 41.7%) was dominated by the term *coagulation* (87/123, 70.7%), followed by *thick (13/123, 10.6%), light (11/123, 8.9%)*, and *watery (8/123, 6.5%)*. Less common were grainy clumps (4/123, 3.3%), thin (4/123, 3.3%), gloopy (2/123, 1.6%), coffee ground inclusions (2/123, 1.6%), slimy (2/123, 1.6%), sticky (2/123, 1.6%), tissuey flakes (2/123, 1.6%), *chunky (1/123, 0.8%), pasty (1/123, 0.8%)*, and others.

Smell (23/295, 7.8%) was less frequently addressed but included terms such as *unpleasant (9/23, 39.1%), irregular (4/23, 17.4%)*, *metallic (1/23, 4.3%), sour (1/23, 4.3%), sweet (2/23, 8.7%),* and *yeast-like (1/23, 4.3%)*. Other appearance-related descriptors included *spotting*, *irregular periods*, and *missed periods*.

#### Appearance Descriptions by Post Type

**Figure 3.**
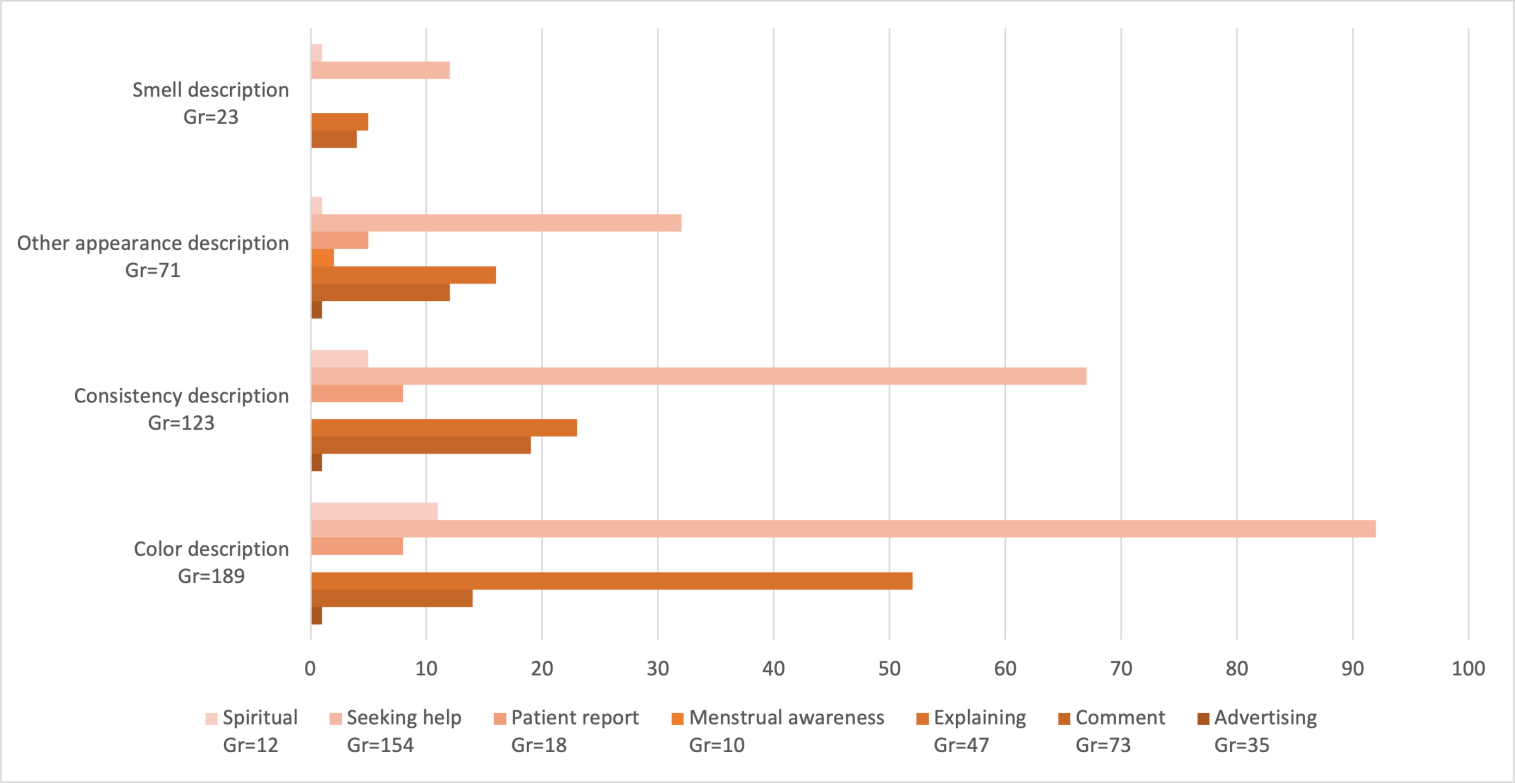
Distribution of qualitative posts describing the appearance of menstrual blood across different post types: visualization of how descriptors of MB appearance (color, consistency, smell, and other) were distributed across post types. Gr: groundedness=number of all quotations coded by a specific code.

Of the quotations coded with *colour-*related descriptions, nearly half occurred in *seeking help* posts (92/189, 48.7%). The second largest share of color descriptions appeared in *explaining* posts (52/189, 27.5%), followed by *comment* posts (14/189, 7.4%). A similar pattern was observed for quotations describing MB *consistency: seeking help* posts accounted for the majority of instances (67/123, 54.5%), followed by *explaining* (23/123, 18.7%) and *comment* posts (19/123, 15.4%). Descriptions of *smell* were predominantly found in *seeking help* posts (12/23, 52.2%). Figure 3 illustrates the distribution of posts describing MB appearance characteristics across post types.

#### Association With Specific Health Conditions

**Figure 4.**
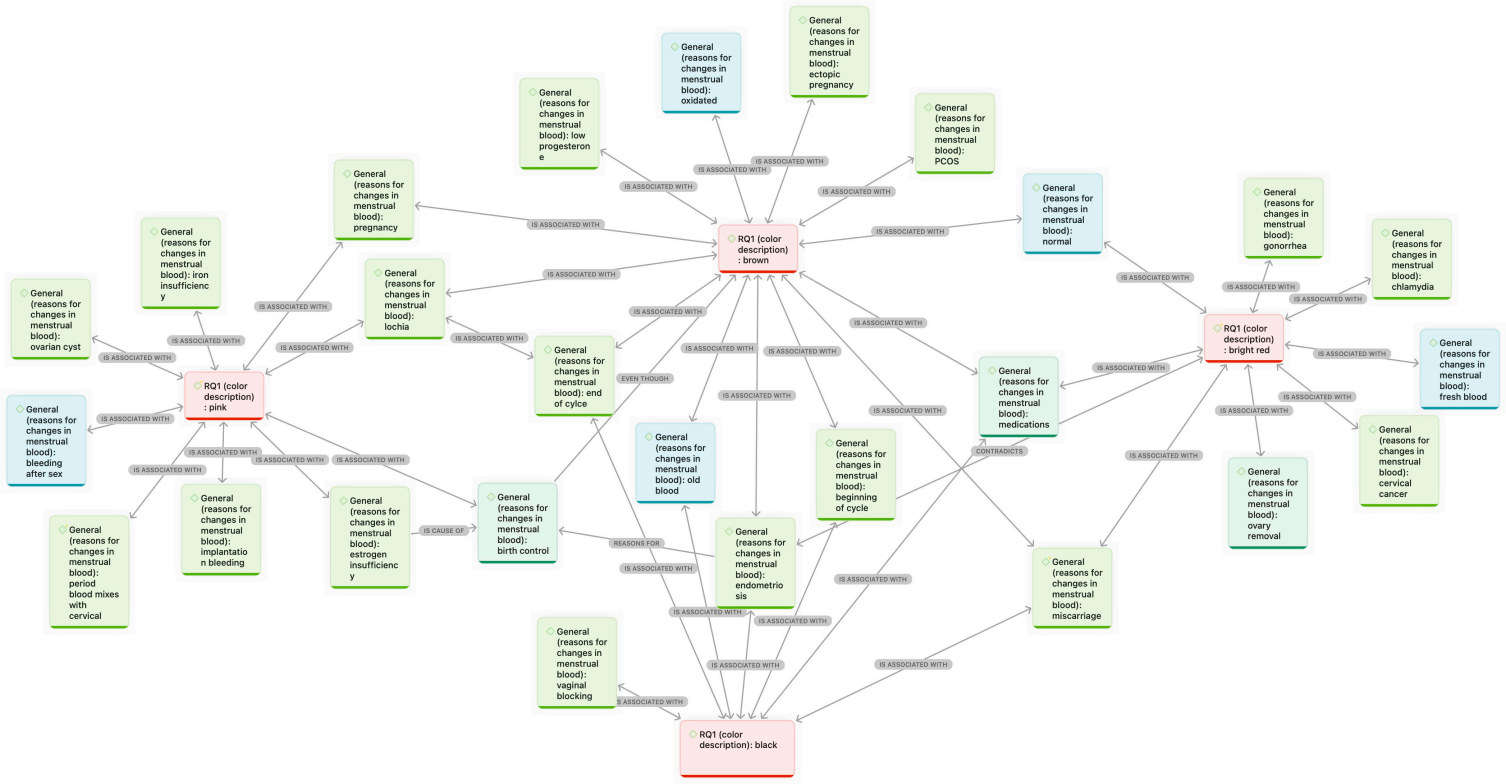
Co-occurrence network linking menstrual blood color descriptors with perceived health conditions: conceptual map illustrating the association between the most frequent color descriptions (brown, bright red, pink, and black) and perceived health conditions mentioned by users. This figure represents perceived, not clinically validated, associations based on user discourse. The red, centered nodes represent color descriptions, the green nodes represent identified medical conditions that influence the appearance, and the turquoise nodes represent other major influencing factors. Arrows indicate the nature of the relationships, with labels specifying associations (eg, “is associated with,” “is cause of,” “contradicts,” and “even though”).

A network analysis was conducted to explore the relationship between MB appearance and health conditions perceived by social media users. The analysis focused on *coagulation* and the 4 most frequently mentioned colors—*brown, bright red, pink*, and *black*. These were analyzed in relation to the code group *reasons for changes in MB*, excluding codes that were rare or unrelated. A visualization of both network analyses is presented in Figures4  5.

**Figure 5.**
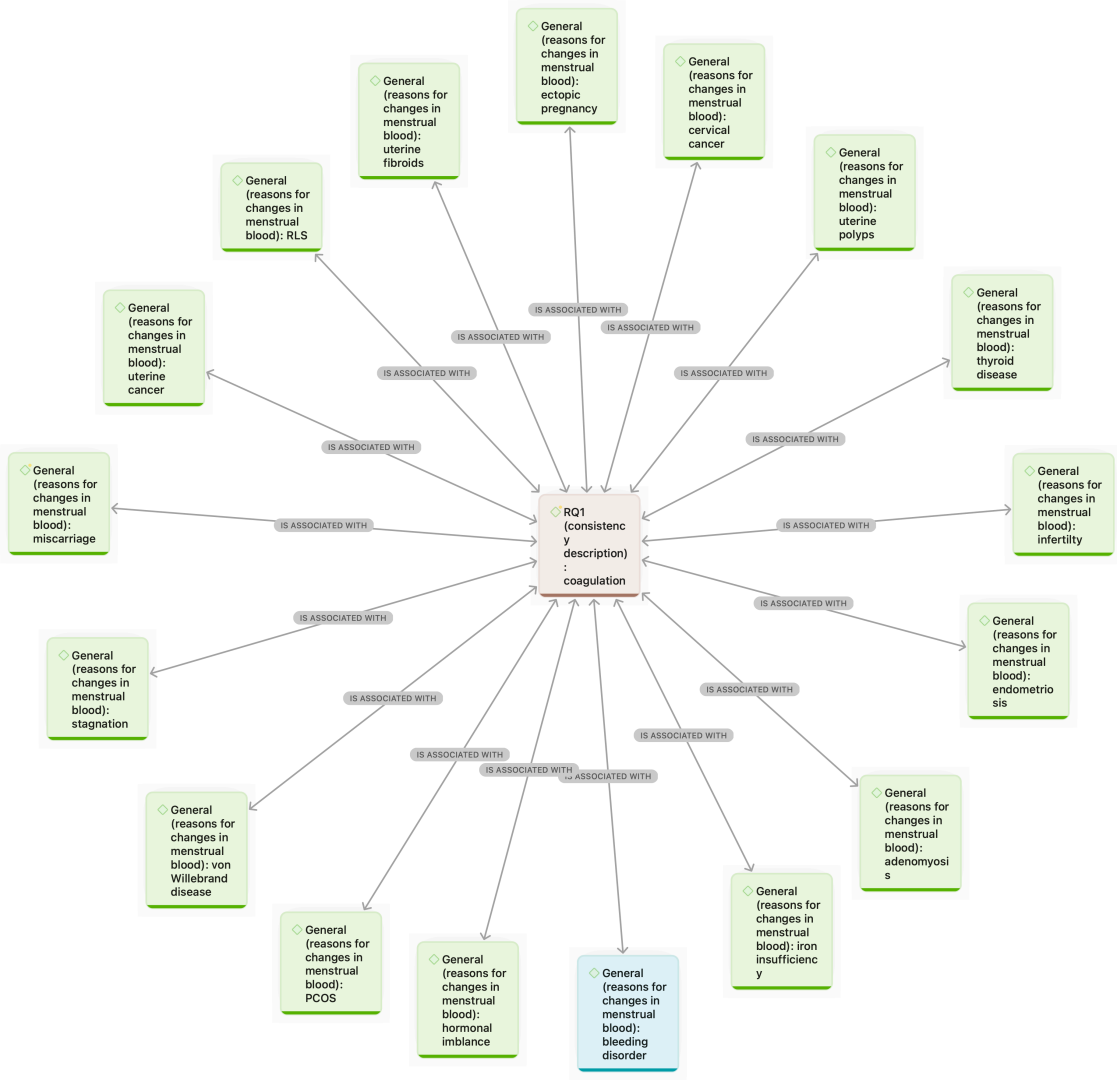
Co-occurrence network linking menstrual blood consistency descriptors with perceived health conditions: conceptual map illustrating relationships between coagulation and associated health conditions mentioned in user posts. The red, centered node represents consistency description, the green nodes represent identified medical conditions that influence the appearance, and the turquoise nodes represent other major influencing factors. Arrows indicate the nature of the relationships, with labels specifying associations (eg, “is associated with,” “is cause of,” “contradicts,” and “even though”).

*Brown* MB was linked to conditions such as *low progesterone*, *ectopic pregnancy*, *PCOS* (polycystic ovary syndrome), *lochia*, *endometriosis*, *miscarriage*, and the *use of medication*. It was also associated with normal phases of the menstrual cycle (eg, *oxidized blood* or the *start and end of menstruation*). Notably, some individuals reported experiencing *brown* MB despite not expecting to menstruate due to contraceptive use. *Pink* blood was connected to *hormonal contraceptives*, *low estrogen*, *iron deficiency*, *ovarian cysts*, *implantation bleeding*, and *mixed with cervical fluid. Bright red* blood was often mentioned in relation to *miscarriage*, *cervical cancer*, *sexually transmitted infections* (eg, gonorrhea and chlamydia), and *surgery* (eg, ovary removal). *Black* MB appeared in relation to *endometriosis*, *miscarriage*, *medication use*, and *vaginal obstruction*, as well as *typical menstrual transitions*.

*Coagulation* was linked to both clinical and cyclical causes, including *uterine polyps*, *adenomyosis*, *endometriosis*, *thyroid issues*, *PCOS*, *hormonal imbalance*, and *uterine cancer*, along with factors like *medication use* and *menstrual phase*.

### RQ2: Qualitative and Quantitative Perception of MB as a Diagnostic Tool

In total, 80 posts addressing the diagnostic potential of MB (RQ2) were coded with a set of 42 codes in 7 code groups (*benefits*, *companies*, *hopes for the future*, *concerns*, *diagnostic methods*, *Research*, and *Other*) in 115 quotations.

#### Companies and Research Initiatives

**Figure 6.**
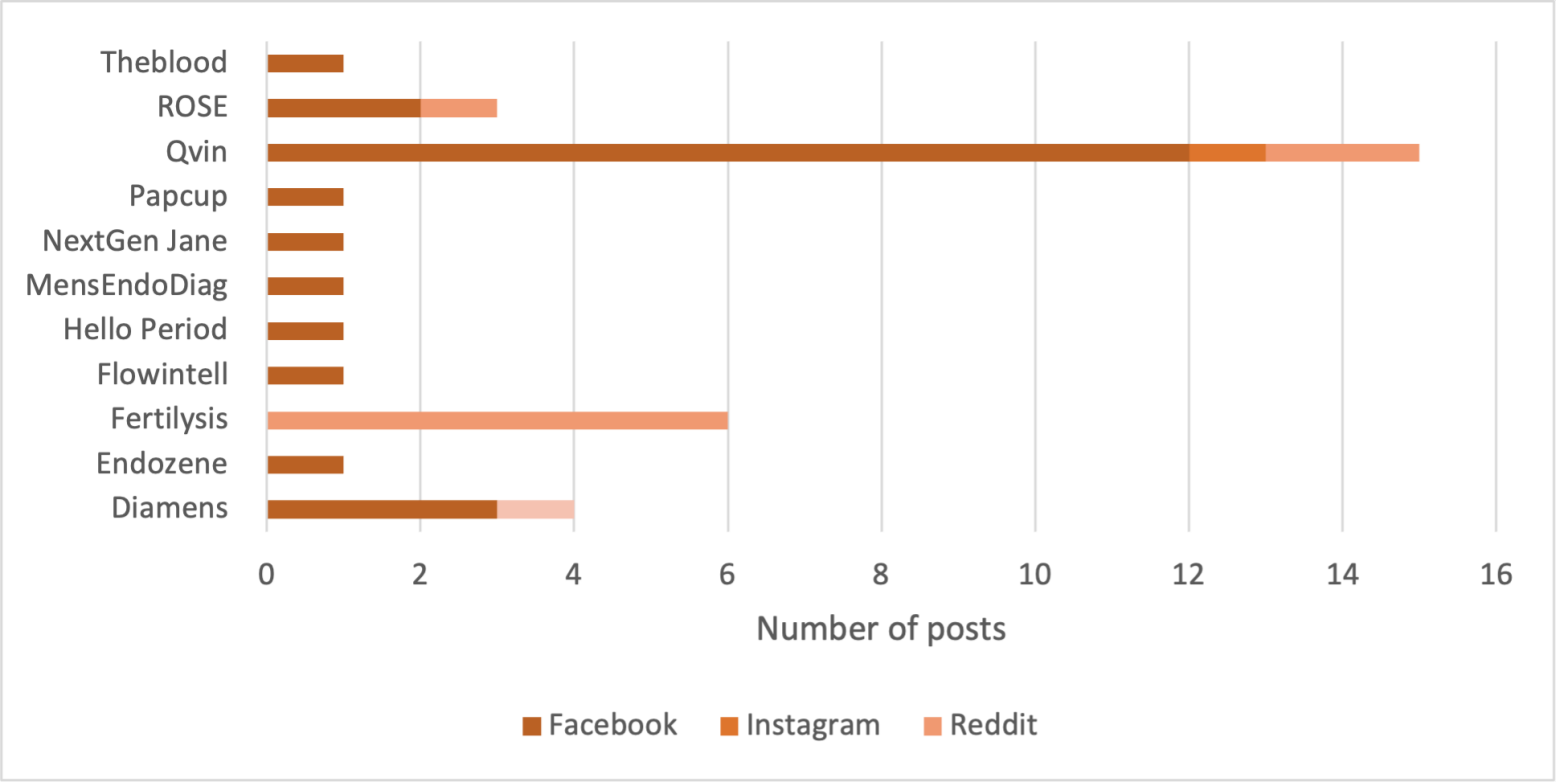
Mentions of diagnostic companies and technologies across platforms: bar chart showing the number of posts mentioning diagnostic companies or research groups related to the use of menstrual blood for diagnostic purposes across 3 social media platforms (Facebook, Instagram, and Reddit).

To assess how MB is discussed as a diagnostic tool, we identified 11 different companies and research groups, which were named 34 times in social media posts (n=80). Frequently mentioned organizations were *Qvin* (15/34, 44.1%) and *Fertilysis* (6/34, 17.6%). Other commercial entities mentioned less frequently included *Hello Period* (1/34, 2.9%), *Theblood* (1/34, 2.9%), *Diamens* (3/23, 8.8%), and *Papcup* (1/34, 2.9%). In addition, users referred to several research-oriented initiatives, including *ROSE* (3/23, 8.8%), *NextGen Jane* (1/34, 2.9%), *MensEndoDiag* (1/34, 2.9%), and *Flowintell* (1/34, 2.9%). The distribution is illustrated in Figure 6.

#### Sentiment Analysis

All quotations discussing diagnostic use of MB (n=115) were designated as positive (n=110), negative (n=19), or neutral (n=16), with the possibility of overlapping (eg, when expressing both positive feelings and concerns). Most posts expressed positive sentiment (110/115, 95.7%), indicating strong support and optimism for innovation in menstrual health. Neutral quotations (16/115, 13.9%) were mainly descriptive, while only a few users expressed negative feelings (19/115, 16.5%), with skepticism or concerns.

#### Perceived Benefits and Concerns

Table 2 summarizes 60 mentions of potential benefits, contrasted by 20 concerns. Additionally, 10 posts expressed surprise at the delayed exploration of the potential of MB.

**Table 2.** Perceived benefits and concerns regarding the use of menstrual blood for diagnostics: summary of qualitative themes describing users’ perceived advantages (60 mentions) and concerns (19 mentions) about potential menstrual blood–based diagnostic tools. Themes were derived from 80 relevant posts.

Perception of theme	Count, n
Benefits	
Destigmatization	2
Early detection	2
Easily accessible	15
Fast	4
Home-based	12
Less intimidating	1
No ethical concerns	1
Noninvasive	10
Pain-free	3
Plentiful	1
Priceworthy	6
Therapeutic use	3
Total	60
Concerns	
Being sold off	1
Collection of MB	1
Expensive	2
Fewer stem cells than in PB	1
Losing body autonomy	2
Menopause or no MB	3
Not getting paid	2
Political climate	1
Rather be tested by a doctor	1
Stigma	3
Toxic if consumed	1
Long time until accessible for all women	1
Yuck factor	1
Total	20

## Discussion

### Principal Findings

This study examined how individuals discuss MB, its appearance, and diagnostic potential on social media. Discussions of MB appearance were primarily framed within help-seeking contexts, particularly on Facebook and Reddit, where users asked whether variations in color (eg, brown, red, or pink) and texture (eg, coagulation) should be considered normal. Alongside these questions, explanatory posts interpreted such variations as physiological and within the range of typical menstrual experiences. Discussions addressing the diagnostic potential of MB were comparatively limited; however, while concerns regarding the costs or not experiencing menstrual bleeding were expressed, the overall sentiment toward the prospective diagnostic use of MB was largely positive.

The predominance of help-seeking posts, followed by explanatory contributions, suggests that social media platforms function as alternative spaces for menstrual health inquiry and social support [33]. Previous research has shown that social media plays a significant role in individual well-being and can both benefit and harm users, depending on how these platforms are used [34]. While social comparison and isolation may negatively affect well-being, meaningful interactions, such as the exchange of personal menstrual health experiences, have the potential to foster social support and improve both general well-being and menstrual health in particular [33].

Regarding the accuracy of menstrual health information shared, verification remains challenging. Although licensed health care professionals may disseminate medical advice on social media platforms, it is often difficult for users to distinguish between self-identified “experts” and credentialed professionals. This ambiguity raises concerns about the reliability of menstrual health advice encountered online and highlights a potential gap in accessible, authoritative menstrual health communication. As a result, individuals may increasingly rely on peer-generated or unverifiable information to address menstrual health questions. Thus, health professionals need to be aware of the influence of social media on health decision-making [4,17].

Even though the medical accuracy of menstrual health advice shared on social media cannot be verified, users actively draw connections between specific MB appearances and perceived pathologies or physiological menstrual cycle changes. To date, scientific research and clinical guidelines have largely focused on menstrual cycle characteristics, such as cycle length, bleeding duration, and volume, while neglecting the appearance of MB itself [35-38]. Against this background, the associations observed in social media discourse show notable thematic overlap with the limited scientific literature. Beyond this, most explanations available to the public stem from a wide range of clinical websites (eg, Cleveland Clinic) but do not offer systematic empirical analysis [39].

The findings of our study indicate that descriptions of MB appearance on social media predominantly focus on colors. While brown, bright red, pink, and black were the most frequently described colors, a wide spectrum of additional color variations was also reported. Analysis by Musarofah and Mahmudah [40] of the 2 historical manuscripts “*Serat Piwulang Estri*” and “*Risalah Al-Mahîd*” described specific MB colors as indicators of weak (eg, yellow and gray) or strong (eg, blackish red and bright red) health, suggesting an early conceptual link between MB color and well-being. Similar patterns emerged in this study. Social media users in this study often associated bright red MB with adverse outcomes of physiological changes, but also with miscarriage, cervical cancer, or sexually transmitted diseases. Darker shades, such as brown, were described both in relation to potential health concerns and as normal variations of the menstrual cycle, explained by prolonged retention of blood in the uterus. An article of Cleveland Clinic shows similar interpretations of bright red (fresh and healthy) and dark red (old and oxidized) blood [39]. Comparable associations between MB color and reproductive health have also been reported by Xiping et al [41], who found that bright red MB was associated with significantly lower pregnancy rates and light red MB with an increased risk of miscarriage. Both Musarofah and Mahmudah [40] and social media users associated pink MB with low estrogen levels. Cleveland Clinic, however, associated pink MB with normal color appearance during the first day of a period [39].

Notably, although 53.8% (1135/2109) of participants in the study by Xiping et al [41] reported blood coagulation, no increased risk of infertility or miscarriage was observed. However, they reported various conditions, such as uterine polyps, uterine fibroids, endometriosis, adenomyosis, and hormonal imbalances in connection to coagulation, all of which were also linked by social media users to MB clots. According to the Cleveland Clinic, the size of a blood clot is the distinguishing factor between physiological coagulation and pathological changes [39]. These findings highlight the potential of drawing menstrual health information from the appearance of MB. However, due to limited literature, there is a need for systematic research into MB color and consistency characteristics.

Smell was rarely discussed, but when mentioned, it was framed as unusual or concerning. Research on MB odor is scarce; however, parallels can be drawn from clinical descriptions of vaginal discharge, where specific odors may accompany infections, as outlined in standard gynecology resources [42-44].

Discussion about the diagnostic use of MB was relatively limited in social media discourse (80 posts compared to 243 posts discussing MB’s appearance characteristics). Zaheer et al [11] similarly reported limited public awareness of MB’s biomedical potential. However, when present, it was predominantly framed in a positive and anticipatory manner. A limited, yet positive awareness of the potential of MB was also shown by Kaur et al [45], who reported that only 28.3% (141/499) of participants knew about the possibility of donating MB, but 64.7% (323/499) of participants saw benefits for society in doing so. Manley et al [46] found that 78% (80/102) of menstruating individuals were open to donating MB for stem cell research, with many reporting a more positive perception of their menstrual cycle following participation. Notably, women affected by menstrual disorders, especially endometriosis, are particularly willing to donate MB for research purposes [47], suggesting that populations most affected by menstrual morbidity may also be most receptive to MB-based diagnostics for personal health monitoring.

Recent technological developments illustrate this growing interest; companies such as *Qvin* [48] developed home-based MB collection devices for biomarker analysis, including hemoglobin A1c, while *Fertilysis* [49] is exploring MB-based diagnostics relevant to fertility and hormonal assessment. AI-supported tools, such as *MenstruAI* [14], further show how computational approaches may complement biological assays in future menstrual health diagnostics. Advantages, such as noninvasive sampling, easy access to the sample, and having the possibility of doing home-based sampling, expressed by social media users closely mirrored those described in scientific literature. Frequently cited benefits included the noninvasiveness of MB collection, its ease of access, home-based self-collection, and the potential elimination of discomfort associated with conventional diagnostic procedures [11]. At the same time, users raised concerns related to body autonomy, lack of financial compensation for MB donation, logistical challenges, such as transportation and processing, and the exclusion of individuals who do not menstruate, due to menopause or hormonal disorders. Similar limitations have been reported by Zaheer et al [11], including restricted sample volume, hormonal fluctuations, contamination risks, interference from menstrual products, stigma, and the missing menstrual bleeding of postmenopausal individuals, children, or individuals experiencing menstrual disorders.

Practical feasibility emerged as a central issue. Menstrual cups are widely reported as an acceptable and effective method for noninvasive MB collection [47,50,51], yet MB can only be collected once per menstrual cycle and within a limited time window, posing challenges particularly for individuals with irregular cycles, infertility, or amenorrhea [10]. Despite growing interest, no standardized clinical protocols currently exist for the collection, transport, processing, and storage of MB samples [10,11]. Nevertheless, emerging research highlights substantial diagnostic and therapeutic potential. MB offers alternative approaches for cervical cancer screening, the diagnosis of endometriosis, genital tuberculosis, diabetes mellitus, altered lipid profiles, and sexually transmitted diseases. Furthermore, due to their pluripotency and low immunogenicity, menstrual blood–derived stem cells have shown promising results in regenerative and therapeutic applications, including the treatment of infertility, premature ovarian failure, intrauterine adhesions, chronic liver disease, and musculoskeletal injuries [15].

These results suggest a marked gap between public interest and clinical implementation. Nonetheless, SML offers valuable insights into lived experiences, especially when clinical engagement is limited or absent. To our knowledge, this is the first study to look at MB discourse, revealing that many individuals already use the appearance of MB as an informal health indicator, particularly in contexts of diagnostic uncertainty or lack of medical recognition. Other social listening studies have already explored the potential of analyzing online discussions for gaining insights into societal or patients’ behaviors. Based on this potential, we extended the insights into how individuals experience menstrual bleeding and are open to further establishment of using MB for diagnostics.

Future research should investigate the diagnostic potential of MB, focusing on visual features, and explore how menstrual knowledge is shaped across time, cultures, and biomedical contexts. Moreover, awareness campaigns are needed to bridge the gap between public interest and clinical application [45]. Engaging both patients and health care providers is essential to overcoming stigma and improving diagnostic pathways.

### Limitations

This study is subject to several limitations that should be considered when interpreting the findings. First, although the analysis focused on the “appearance” of MB, only textual descriptions could be included because images and videos could not be reliably analyzed. Excluding these visual materials means that the findings for RQ1 are based entirely on users’ self-reported descriptions rather than the appearance itself. This introduces an interpretive gap between users’ observations and their articulation, reducing the precision with which appearance-related interpretations can be understood. A second limitation arises from the short extraction period and technical constraints due to downloading restrictions from Mention and Apify. Apify hashtag scrapers retrieved only the most recent 100 posts per hashtag, and Mention alerts captured content within predefined retrospective and prospective windows. As a result, the dataset should be interpreted as a snapshot of the public discourse of all MB-related discussions online. Posting activity patterns, platform algorithms, and seasonal or event-driven fluctuations may therefore influence the types of posts retrieved.

Third, only English-language posts were included to ensure analytic consistency and feasibility. This choice inevitably skews the dataset toward English-speaking users and may underrepresent cultural or regional differences in how MB is discussed, interpreted, and linked to health conditions. Fourth, social media primarily reflects the perspectives of younger, digitally active users [52]; the findings may therefore not fully represent the broader population, particularly individuals without consistent internet access or those less likely to engage in public online discourse. Additional limitations inherent to SML also apply. Due to restricted data access, limited sample size, and the lack of standardized protocols, bias control and representativeness remain challenging.

### Conclusion

This study demonstrates that individuals increasingly turn to social media platforms to seek information and reassurance about MB appearance, underscoring a gap between the demand for evidence-based menstrual health information and the limited scientific literature addressing MB characteristics. The prominence of peer-guided explanations highlights both unmet informational needs and users’ openness to engaging with menstrual health topics outside clinical settings. Together with the largely positive attitudes toward the potential diagnostic use of MB, these findings emphasize the need to expand research on MB characteristics and to systematically evaluate its diagnostic potential for menstrual health–related conditions.

## Supplementary material

10.2196/85550 Multimedia Appendix 1 Search strategies.

## Abbreviations

AI: artificial intelligence
MB: menstrual blood
PCOS: polycystic ovary syndrome
RQ: research question
SML: social media listening
