# Supplementary material for: Interpretations of Menstrual Blood Appearance and Diagnostic Potential Among Social Media Users: Cross-Sectional Mixed Methods Social Media Listening Study
Source: J Med Internet Res. 2026 May 4;28:e85550. doi: 10.2196/85550 (PMC13138715; doi:10.2196/85550)
Supplement: Multimedia Appendix 1 [file jmir-v28-e85550-s001.docx]

# Supplementary material

[Table S1: Keyword Search on Facebook and Reddit 1](#_Toc221096227)

[Table S2: Reddit results; inclusion and exclusion criteria 4](#_Toc221096228)

[Table S3: Facebook results; inclusion and exclusion criteria 5](#_Toc221096229)

[Table S4: Downloaded posts 6](#_Toc221096230)

[Table S5: Hashtag results in Instagram and TikTok 6](#_Toc221096231)

[Table S6: Codebook 8](#_Toc221096232)

[Table S7: Distribution of post types across social media platforms (Facebook, Instagram, Reddit, and TikTok) with the number of posts and respective percentages of total platform-specific mentions 11](#_Toc221096233)

1. Search strategies

S1.1. Group Search

For both Facebook and Reddit, four keywords were identified:” Menstrual Blood”,” Periods”, ”Period Blood”, and ”Endometriosis.” The first ten groups (on Facebook) and communities (on Reddit) were listed along with their member counts. Of these forty groups on Facebook, seven groups appeared across multiple keyword searches. One private group was excluded on Facebook. Six groups were screened for relevance, resulting in the inclusion of three groups in the analysis. Of the 40 communities on Reddit, six groups appeared in multiple keyword searches. After screening for relevance only one community was included in the analysis. By using the ’Facebook Group Scraper’ and ’Reddit Scraper Lite’ of Apify, the top posts of the four included groups were downloaded, resulting in 350 posts (200 posts from Facebook groups, 150 posts from Reddit communities).

Table S1: Keyword Search on Facebook and Reddit

| Source | Search Term | Group Nr. | Group name | Members | Note | Posts |
| --- | --- | --- | --- | --- | --- | --- |
| Reddit | Menstrual Blood | 1 | r/scat_lifegetsSHITTY | 6.500 | 18+ |  |
| Reddit | Menstrual Blood | 2 | r/menstrualpainting | 30 |  |  |
| Reddit | Menstrual Blood | 3 | r/menstrualcups | 50.000 |  |  |
| Reddit | Menstrual Blood | 4 | r/badwomensanatomy | 573.000 |  |  |
| Reddit | Menstrual Blood | 5 | r/mindfulMensis | 390 |  |  |
| Reddit | Menstrual Blood | 6 | r/todayilearned | 40.000.000 |  |  |
| Reddit | Menstrual Blood | 7 | r/Periods | 88.000 |  |  |
| Reddit | Menstrual Blood | 8 | r/WTF | 7.100.000 |  |  |
| Reddit | Menstrual Blood | 9 | r/TwoXChromosomes | 14.000.000 |  |  |
| Reddit | Menstrual Blood | 10 | r/TrollXChromosomes | 835.000 |  |  |
| Facebook | Menstrual Blood | 1 | Girl Talk With Dr. Amber: Menstrual Problems + Solution.Period. | 28.300 |  | 8 posts a month |
| Facebook | Menstrual Blood | 2 | The nub tech | 7.400 |  | 10+ posts a day |
| Facebook | Menstrual Blood | 3 | How to solve your menstrual problem | 29.000 |  | 2 posts a day |
| Facebook | Menstrual Blood | 4 | Menstrual Blood | 3 |  |  |
| Facebook | Menstrual Blood | 5 | Pregnancy Tips and Pregnancy related topics | 610.000 |  | 10+ posts a day |
| Facebook | Menstrual Blood | 6 | The True History of the Rh Negative Blood Groups | 10.000 |  | 2 posts a day |
| Facebook | Menstrual Blood | 7 | Cloth Menstrual Pads: Buy, Sell And Trade | 7.800 |  |  |
| Facebook | Menstrual Blood | 8 | Treatment consulation: Menstrual and hormonal disorders -Elicare | 8 |  | 2 posts a year |
| Facebook | Menstrual Blood | 9 | ELICARE MENSTRUAL CARE - PHILIPPINES | 230 |  | 10 posts a year |
| Facebook | Menstrual Blood | 10 | OVULATION, MENSTRUAL CYCLE, FERTILITY PROBLEM DISCUSSION | 452 |  | 8 posts a year |
|  |  |  |  |  |  |  |
| Reddit | Periods | 1 | r/Periods | 88.000 |  |  |
| Reddit | Periods | 2 | r/MenAskAboutPeriods | 61 |  |  |
| Reddit | Periods | 3 | r/period_thoughts | 2.600 |  |  |
| Reddit | Periods | 4 | r/TwoChromosomes | 14.000.000 |  |  |
| Reddit | Periods | 5 | r/AskWomen | 5.500.000 | 18+ |  |
| Reddit | Periods | 6 | r/PeriodStories | 769 |  |  |
| Reddit | Periods | 7 | r/ImportantPeriods | 11 |  |  |
| Reddit | Periods | 8 | r/TrollXChromosomes | 835.000 |  |  |
| Reddit | Periods | 9 | r/AskReddit | 52.000.000 |  |  |
| Reddit | Periods | 10 | r/VictorianSluts | 72.000 | 18+ |  |
| Facebook | Periods | 1 | Girl Talk With Dr. Amber: Menstrual Problems + Solution.Period. | 28.300 |  | 8 posts a month |
| Facebook | Periods | 2 | PERIOD PROBLEM | 110.000 |  | 10 posts a day |
| Facebook | Periods | 3 | Menstruation Periods and How to Handle | 32.000 |  | 8 posts a month |
| Facebook | Periods | 4 | How to restore your period bacl | 236.000 |  | 9 posts a day |
| Facebook | Periods | 5 | Menstruation (Period) share problem | 28.000 |  | 10+ posts a day |
| Facebook | Periods | 6 | PERIOD PROBLEM & PREGNANCY PROBLEM | 73 |  | 9 posts a year |
| Facebook | Periods | 7 | Ovaulation and period track | 1.400 |  | 4 posts a month |
| Facebook | Periods | 8 | Period Tips & Tricks for Middle School girls and their parents | 2.900 |  |  |
| Facebook | Periods | 9 | Period pain | 1.400 |  | 5 posts a month |
| Facebook | Periods | 10 | Period Problems | 31.000 |  |  |
|  |  |  |  |  |  |  |
| Reddit | Period Blood | 1 | r/Periods | 88.000 |  |  |
| Reddit | Period Blood | 2 | r/camphalfblood | 166.000 |  |  |
| Reddit | Period Blood | 3 | r/PeriodPanties | 13.000 | 18+ |  |
| Reddit | Period Blood | 4 | r/TrollCXhromosomes | 835.000 |  |  |
| Reddit | Period Blood | 5 | r/BloodBlister | 13 |  |  |
| Reddit | Period Blood | 6 | r/sasaeng | 125 |  |  |
| Reddit | Period Blood | 7 | r/badwomensanatomy | 573.000 |  |  |
| Reddit | Period Blood | 8 | r/AskWomen | 5.500.000 | 18+ |  |
| Reddit | Period Blood | 9 | r/WTF | 7.100.000 |  |  |
| Reddit | Period Blood | 10 | r/Wellworn | 315.000 |  |  |
| Facebook | Period Blood | 1 | Girl Talk With Dr. Amber: Menstrual Problems + Solution.Period. | 28.300 |  | 8 posts a month |
| Facebook | Period Blood | 2 | PERIOD PROBLEM | 110.000 |  | 10 posts a day |
| Facebook | Period Blood | 3 | Ovaulation and period track | 1.400 |  | 4 posts a month |
| Facebook | Period Blood | 4 | how to calculate period and ovulation and also how to get pregnant fast | 54.000 |  | 2 posts a day |
| Facebook | Period Blood | 5 | How to restore your period bacl | 236.000 |  | 9 posts a day |
| Facebook | Period Blood | 6 | Menstruation (Period) share problem | 28.000 |  | 10+ posts a day |
| Facebook | Period Blood | 7 | PERIOD PROBLEM & PREGNANCY PROBLEM | 73 |  | 9 posts a year |
| Facebook | Period Blood | 8 | How To Handle Menstruation Periods | 1.400 |  | 9 posts a year |
| Facebook | Period Blood | 9 | Menstruation Periods and How to Handle | 32.000 |  | 8 posts a month |
| Facebook | Period Blood | 10 | healing for period pain an block tubes | 1.700 |  | 10 posts a year |
|  |  |  |  |  |  |  |
| Reddit | Endometriosis | 1 | r/endometriosis | 90.000 |  |  |
| Reddit | Endometriosis | 2 | r/NaturalEndometriosis | 366 |  |  |
| Reddit | Endometriosis | 3 | r/endometriosis_stage4 | 357 |  |  |
| Reddit | Endometriosis | 4 | r/Endo | 76.000 |  |  |
| Reddit | Endometriosis | 5 | r/TwoXChromosomes | 14.000.000 |  |  |
| Reddit | Endometriosis | 6 | r/endometriosis_corner | 1.900 |  |  |
| Reddit | Endometriosis | 7 | r/EndoEnts | 801 |  |  |
| Reddit | Endometriosis | 8 | r/hysterectomy | 31.000 |  |  |
| Reddit | Endometriosis | 9 | r/ChronicPain | 124.000 |  |  |
| Reddit | Endometriosis | 10 | r/endometriosisindia | 195 |  |  |
| Facebook | Endometriosis | 1 | Endometriosis Support Group | 76.000 |  | 10+ posts a day |
| Facebook | Endometriosis | 2 | Holistic Adenomyosis and Endometriosis Community Support | 8.200 |  | 4 posts a day |
| Facebook | Endometriosis | 3 | Endometriosis Supprt Group | 99.000 |  | 10+ posts a day |
| Facebook | Endometriosis | 4 | Endometriosis Awareness & Support Group India #ENDOCHAMPIONS | 341 |  | 5 posts a month |
| Facebook | Endometriosis | 5 | Endometriosis and Infertility Awareness Support Group | 549 |  |  |
| Facebook | Endometriosis | 6 | Endometriosis support group | 4.800 |  | 10 posts a day |
| Facebook | Endometriosis | 7 | Grupo de apoyo mujeres con endometriosis interior del pais | 242 |  | 20 posts a year |
| Facebook | Endometriosis | 8 | PCOS & Endometriosis Community | 495 |  |  |
| Facebook | Endometriosis | 9 | Endometriosis Supprt SA (Closed Group) | 3.400 |  | 3 posts a day |
| Facebook | Endometriosis | 10 | The Endo Space -Endometriosis Supprt Group | 19.000 |  | 10+ posts a day |

Table S2: Reddit results; inclusion and exclusion criteria

| Source | Search Term | Group Nr. | Group name | Members | Note | Duplicates | Exclusion/Inclusion |
| --- | --- | --- | --- | --- | --- | --- | --- |
| Reddit | Periods | 9 | r/AskReddit | 52.000.000 |  |  |  |
| Reddit | Periods | 5 | r/AskWomen | 5.500.000 | 18+ | D | not relevant |
| Reddit | Period Blood | 8 | r/AskWomen | 5.500.000 | 18+ | D | not relevant |
| Reddit | Menstrual Blood | 4 | r/badwomensanatomy | 573.000 |  | D | not relevant |
| Reddit | Period Blood | 7 | r/badwomensanatomy | 573.000 |  | D | not relevant |
| Reddit | Period Blood | 5 | r/BloodBlister | 13 |  |  |  |
| Reddit | Period Blood | 2 | r/camphalfblood | 166.000 |  |  |  |
| Reddit | Endometriosis | 9 | r/ChronicPain | 124.000 |  |  |  |
| Reddit | Endometriosis | 4 | r/Endo | 76.000 |  |  |  |
| Reddit | Endometriosis | 7 | r/EndoEnts | 801 |  |  |  |
| Reddit | Endometriosis | 1 | r/endometriosis | 90.000 |  |  |  |
| Reddit | Endometriosis | 6 | r/endometriosis_corner | 1.900 |  |  |  |
| Reddit | Endometriosis | 3 | r/endometriosis_stage4 | 357 |  |  |  |
| Reddit | Endometriosis | 10 | r/endometriosisindia | 195 |  |  |  |
| Reddit | Endometriosis | 8 | r/hysterectomy | 31.000 |  |  |  |
| Reddit | Periods | 7 | r/ImportantPeriods | 11 |  |  |  |
| Reddit | Periods | 2 | r/MenAskAboutPeriods | 61 |  |  |  |
| Reddit | Menstrual Blood | 3 | r/menstrualcups | 50.000 |  |  |  |
| Reddit | Menstrual Blood | 2 | r/menstrualpainting | 30 |  |  |  |
| Reddit | Menstrual Blood | 5 | r/mindfulMensis | 390 |  |  |  |
| Reddit | Endometriosis | 2 | r/NaturalEndometriosis | 366 |  |  |  |
| Reddit | Periods | 3 | r/period_thoughts | 2.600 |  |  |  |
| Reddit | Period Blood | 3 | r/PeriodPanties | 13.000 | 18+ |  |  |
| Reddit | Menstrual Blood | 7 | r/Periods | 88.000 |  | D | included |
| Reddit | Periods | 1 | r/Periods | 88.000 |  | D | included |
| Reddit | Period Blood | 1 | r/Periods | 88.000 |  | D | included |
| Reddit | Periods | 6 | r/PeriodStories | 769 |  |  |  |
| Reddit | Period Blood | 6 | r/sasaeng | 125 |  |  |  |
| Reddit | Menstrual Blood | 1 | r/scat_lifegetsSHITTY | 6.500 | 18+ |  |  |
| Reddit | Menstrual Blood | 6 | r/todayilearned | 40.000.000 |  |  |  |
| Reddit | Period Blood | 4 | r/TrollCXhromosomes | 835.000 |  | D | not relevant |
| Reddit | Menstrual Blood | 10 | r/TrollXChromosomes | 835.000 |  | D | not relevant |
| Reddit | Periods | 8 | r/TrollXChromosomes | 835.000 |  | D | not relevant |
| Reddit | Periods | 4 | r/TwoChromosomes | 14.000.000 |  | D | not relevant |
| Reddit | Menstrual Blood | 9 | r/TwoXChromosomes | 14.000.000 |  | D | not relevant |
| Reddit | Endometriosis | 5 | r/TwoXChromosomes | 14.000.000 |  | D | not relevant |
| Reddit | Periods | 10 | r/VictorianSluts | 72.000 | 18+ |  |  |
| Reddit | Period Blood | 10 | r/Wellworn | 315.000 |  |  |  |
| Reddit | Menstrual Blood | 8 | r/WTF | 7.100.000 |  | D | not relevant |
| Reddit | Period Blood | 9 | r/WTF | 7.100.000 |  | D | not relevant |

Table S3: Facebook results; inclusion and exclusion criteria

| Source | Search Term | Group Nr. | Group name | Members | Posts | Public/  Privat | Duplicates | Exclusion/  Inclusion |
| --- | --- | --- | --- | --- | --- | --- | --- | --- |
| Facebook | Menstrual Blood | 7 | Cloth Menstrual Pads: Buy, Sell And Trade | 7.800 |  | private |  |  |
| Facebook | Menstrual Blood | 9 | ELICARE MENSTRUAL CARE - PHILIPPINES | 230 | 10 posts a year | public |  |  |
| Facebook | Endometriosis | 5 | Endometriosis and Infertility Awareness Support Group | 549 |  | private |  |  |
| Facebook | Endometriosis | 4 | Endometriosis Awareness & Support Group India #ENDOCHAMPIONS | 341 | 5 posts a month | public |  |  |
| Facebook | Endometriosis | 1 | Endometriosis Support Group | 76.000 | 10+ posts a day | private |  |  |
| Facebook | Endometriosis | 3 | Endometriosis Support Group | 99.000 | 10+ posts a day | private |  |  |
| Facebook | Endometriosis | 6 | Endometriosis support group | 4.800 | 10 posts a day | public |  |  |
| Facebook | Endometriosis | 9 | Endometriosis Support SA (Closed Group) | 3.400 | 3 posts a day | private |  |  |
| Facebook | Menstrual Blood | 1 | Girl Talk With Dr. Amber: Menstrual Problems + Solution.Period. | 28.300 | 8 posts a month | public | D | included |
| Facebook | Periods | 1 | Girl Talk With Dr. Amber: Menstrual Problems + Solution.Period. | 28.300 | 8 posts a month | public | D | included |
| Facebook | Period Blood | 1 | Girl Talk With Dr. Amber: Menstrual Problems + Solution.Period. | 28.300 | 8 posts a month | public | D | included |
| Facebook | Endometriosis | 7 | Grupo de apoyo mujeres con endometriosis interior del pais | 242 | 20 posts a year | public |  |  |
| Facebook | Period Blood | 10 | healing for period pain an block tubes | 1.700 | 10 posts a year | public |  |  |
| Facebook | Endometriosis | 2 | Holistic Adenomyosis and Endometriosis Community Support | 8.200 | 4 posts a day | private |  |  |
| Facebook | Period Blood | 4 | how to calculate period and ovulation and also how to get pregnant fast | 54.000 | 2 posts a day | private |  |  |
| Facebook | Period Blood | 8 | How To Handle Menstruation Periods | 1.400 | 9 posts a year | public |  |  |
| Facebook | Periods | 4 | How to restore your period back | 236.000 | 9 posts a day | private | D | private |
| Facebook | Period Blood | 5 | How to restore your period bacl | 236.000 | 9 posts a day | private | D | private |
| Facebook | Menstrual Blood | 3 | How to solve your menstrual problem | 29.000 | 2 posts a day | public |  |  |
| Facebook | Menstrual Blood | 4 | Menstrual Blood | 3 |  | private |  |  |
| Facebook | Periods | 5 | Menstruation (Period) share problem | 28.000 | 10+ posts a day | public | D | included |
| Facebook | Period Blood | 6 | Menstruation (Period) share problem | 28.000 | 10+ posts a day | public | D | included |
| Facebook | Periods | 3 | Menstruation Periods and How to Handle | 32.000 | 8 posts a month | public | D | not relevant |
| Facebook | Period Blood | 9 | Menstruation Periods and How to Handle | 32.000 | 8 posts a month | public | D | not relevant |
| Facebook | Periods | 7 | Ovaulation and period track | 1.400 | 4 posts a month | public | D | not relevant |
| Facebook | Period Blood | 3 | Ovaulation and period track | 1.400 | 4 posts a month | public | D | not relevant |
| Facebook | Menstrual Blood | 10 | OVULATION, MENSTRUAL CYCLE, FERTILITY PROBLEM DISCUSSION | 452 | 8 posts a year | public |  |  |
| Facebook | Endometriosis | 8 | PCOS & Endometriosis Community | 495 |  | private |  |  |
| Facebook | Periods | 9 | Period pain | 1.400 | 5 posts a month | public |  |  |
| Facebook | Periods | 2 | PERIOD PROBLEM | 110.000 | 10 posts a day | public | D | included |
| Facebook | Period Blood | 2 | PERIOD PROBLEM | 110.000 | 10 posts a day | public | D | included |
| Facebook | Periods | 6 | PERIOD PROBLEM & PREGNANCY PROBLEM | 73 | 9 posts a year | public | D | not relevant |
| Facebook | Period Blood | 7 | PERIOD PROBLEM & PREGNANCY PROBLEM | 73 | 9 posts a year | public | D | not relevant |
| Facebook | Periods | 10 | Period Problems | 31.000 |  | private |  |  |
| Facebook | Periods | 8 | Period Tips & Tricks for Middle School girls and their parents | 2.900 |  | public |  |  |
| Facebook | Menstrual Blood | 5 | Pregnancy Tips and Pregnancy related topics | 610.000 | 10+ posts a day | private |  |  |
| Facebook | Endometriosis | 10 | The Endo Space -Endometriosis Support Group | 19.000 | 10+ posts a day | private |  |  |
| Facebook | Menstrual Blood | 2 | The nub tech | 7.400 | 10+ posts a day | private |  |  |
| Facebook | Menstrual Blood | 6 | The True History of the Rh Negative Blood Groups | 10.000 | 2 posts a day | public |  |  |
| Facebook | Menstrual Blood | 8 | Treatment consulation: Menstrual and hormonal disorders -Elicare | 8 | 2 posts a year | public |  |  |

Table S4: Downloaded posts

| Source | Group name/Community | Members | Apify results |
| --- | --- | --- | --- |
| Reddit | r/Periods | 88.000 | 150 |
| Facebook | Girl Talk With Dr. Amber: Menstrual Problems + Solution.Period. | 28.300 | 100 |
| Facebook | Menstruation (Period) share problem | 28.000 | error |
| Facebook | PERIOD PROBLEM | 110.000 | 100 |

S1.2. Hashtag Search

We conducted a hashtag search on Instagram and Reddit using the following hashtags: #menstrualblood, #periodblood and #periods. From a total of 13,000 results of #menstrualblood on Instagram 57 were downloaded using the Apify ’Instagram Hashtag Scraper’. Accordingly, from 425 results on TikTok 100 were downloaded using the Apify ’TikTok Hashtag Scraper’. For #periodblood 57 of 2,.500 results were downloaded on Instagram, and 100 of 6,303 results were downloaded on TikTok. Last for #periods we downloaded 21 of 792,000 posts on Instagram and 99 of 126,700 results on TikTok.

Table S5: Hashtag results in Instagram and TikTok

| Hashtag | Source | Results | Downloaded | Total |
| --- | --- | --- | --- | --- |
| #menstrualblood | TikTok | 425 | 100 | 100 |
| #menstrualblood | Instagram | 13.000 | 30 videos  27 posts | 57 |
| #periodblood | TikTok | 6.307 | 100 | 100 |
| #periodblood | Instagram | 22.500 | 30 videos  27 posts | 57 |
| #periods | TikTok | 126.700 | 99 | 99 |
| #periods | Instagram | 792.000 | 21 posts | 21 |
|  |  |  |  | **434** |

S1.3. Alert Search

Parallel, we used the social listening tool Mention to create six alerts using various search terms. The searched sources included Instagram, TikTok, Facebook, Reddit and X/Twitter. Five alerts were active for a seven-day period (13/02/2025 - 19/02/2025). Three alerts conducted a retrospective search for a period of two years (02/2023 - 01/2025). For the seven-day period, 1,555 and for the two-year period, 3,874 results were downloaded.

Table S6: Results per period of time and alert

|  | **Alert Nr.** | **Alert 1** | | **Alert 2** | | **Alert 3** | | **Alert 4** | | | **Alert 5** | | **Alert 6** | | |
| --- | --- | --- | --- | --- | --- | --- | --- | --- | --- | --- | --- | --- | --- | --- | --- |
|  | **Alert Name** | **MB and Endo** | | **HS_MB+ Diagnostics** | | **HS_PB and period facts** | | **HS_MB+Endo** | | | **MB +hashtag** | | **MB+colour+ diagnostic** | |  |
|  | **Search Term** | ("menstrual blood" OR "period blood" OR menstrualblood OR periodblood OR menstruation)  AND ("endometriosis" OR diagnostics OR diagnosis OR testing) AND (black OR brown OR back OR pink OR orange OR red OR "dark red" OR purple OR colour OR color OR jelly OR thick OR consistency OR appearance OR coagulation) | | ("menstrual blood" OR "period blood" OR menstrualblood OR periodblood)  AND (black OR brown OR pink OR orange OR red OR "dark red" OR purple OR colour OR color OR jelly OR thick OR consistency OR appearance OR coagulation) AND (diagnostic OR diagosis OR testing OR biomarker OR insecure OR unsure OR insecureties) | | ("period blood" OR "menstrual blood" OR menstrualblood OR periodblood OR period OR menstruation) AND (periodpositivity OR periodtalk OR periodfacts OR menstruationmatters OR periodcup OR menstrualcup OR periodart OR menstrualcycle OR timeofthemonth) | | ("menstrual blood" OR "period blood" OR menstrualblood OR periodblood)  AND (endoblood OR endometriosis OR endometriose OR endo OR PCOS OR diabetes OR diseases OR diagnosis OR periodproblem OR fibroids OR infertility OR endobelly OR "cycle irregularities") | | | ("menstrual blood" OR "menstrual\* blood\*" OR "menstrual-b\*" OR menstruation OR "menstrual-cycle" OR "menstrual phase" OR "period blood" OR "#periodblood" OR "period\* blood\*" OR "#period" OR "#menstrualblood" OR "#menstruation")  AND ("diagnosis" OR "diagnose" OR "colour" OR "color" OR "coloration" OR "black" OR "pink" OR "red" OR "orange" OR "testing" OR "test" OR "biomarker\*" OR "dark red" OR brown OR coagulation) | | (menstrualblood OR "menstrual blood" OR periodblood OR "period blood" OR menstruation OR period OR mensies)  AND (black OR brown OR pink OR orange OR red OR "dark red" OR purple OR colour OR color OR jelly OR thick OR consistency OR appearance OR coagulation)  AND (diagnostic OR diagosis OR testing OR biomarkers OR biomarker) | |  |
|  | **Time period** | **7 days** | **2 years** | **7 days** | **2 years** | **7 days** | **2 years** | | **7 days** | **2 years** | **7 days** | **2 years** | **7 days** | **2 years** |  |
| **Sources** | Instagram | 13 | / | / | / | 643 | 3 | | 15 | / | 203 | / | 18 | / |  |
|  | TikTok | / | / | / | / | 82 | 5 | | / | / | 22 | 1 | / | / |  |
|  | Reddit | 16 | 1151 | / | 81 | / | / | | 8 | 606 | 82 | / | 138 | / |  |
|  | Facebook | 1 | 613 | / | 16 | 17 | / | | 2 | 226 | 22 | / | 14 | / |  |
|  | X/Twitter | / | 207 | / | 12 | 28 | 1 | | 4 | 949 | 210 | 3 | 17 | / |  |
| **Total** | | **30** | **1971** | **/** | **109** | **770** | **9** | | **29** | **1781** | **539** | **4** | **187** | **/** |  |
| **Total**  **7 days** | | **1555** | | | | | | | | | | | | |  |
| **Total**  **2 years** | | **3874** | | | | | | | | | | | | |  |

1. Codebook

Table S7: Codebook

| Code | | | | Groundedness |
| --- | --- | --- | --- | --- |
| Research questions | | | | 548 |
|  | General | | | 517 |
|  |  | reasons for changes in menstrual blood | | 248 |
|  |  |  | ● adenomyosis | 8 |
|  |  |  | ● after birth | 2 |
|  |  |  | ● Alzheimer | 1 |
|  |  |  | ● ankylosing spondylitis | 1 |
|  |  |  | ● bacterial vaginosis | 3 |
|  |  |  | ● beginning of cycle | 14 |
|  |  |  | ● birth control | 23 |
|  |  |  | ● bleeding after sex | 3 |
|  |  |  | ● bleeding disorder | 2 |
|  |  |  | ● bleeding during sex | 1 |
|  |  |  | ● blood when brushing teeth | 1 |
|  |  |  | ● cardiovascular diseases | 1 |
|  |  |  | ● cervical cancer | 8 |
|  |  |  | ● chlamydia | 3 |
|  |  |  | ● Covid | 2 |
|  |  |  | ● diabetes | 20 |
|  |  |  | ● dilator | 1 |
|  |  |  | ● ectopic pregnancy | 3 |
|  |  |  | ● end of cycle | 13 |
|  |  |  | ● endometrial cyst removal | 2 |
|  |  |  | ● endometriosis | 76 |
|  |  |  | ● endometritis | 2 |
|  |  |  | ● oestrogen insufficiency | 7 |
|  |  |  | ● fallopian tube removal | 1 |
|  |  |  | ● feel fatigued or experience weak digestion | 1 |
|  |  |  | ● Fibromyalgia | 1 |
|  |  |  | ● fresh blood | 7 |
|  |  |  | ● going to gym | 1 |
|  |  |  | ● gonorrhoea | 3 |
|  |  |  | ● high oestrogen levels | 2 |
|  |  |  | ● hormonal imbalance | 5 |
|  |  |  | ● HPV | 6 |
|  |  |  | ● hypothyroidism | 2 |
|  |  |  | ● implantation bleeding | 5 |
|  |  |  | ● infection | 6 |
|  |  |  | ● infertility | 7 |
|  |  |  | ● inflammation | 2 |
|  |  |  | ● institial cystitis | 1 |
|  |  |  | ● internal heat | 1 |
|  |  |  | ● iron insufficiency | 3 |
|  |  |  | ● laying or sitting | 1 |
|  |  |  | ● Liver Qi Stagnation in TCM | 1 |
|  |  |  | ● lochia | 4 |
|  |  |  | ● losing weight | 1 |
|  |  |  | ● low progesterone | 2 |
|  |  |  | ● medications | 10 |
|  |  |  | ● menopause | 1 |
|  |  |  | ● miscarriage | 9 |
|  |  |  | ● normal | 24 |
|  |  |  | ● old blood | 15 |
|  |  |  | ● ovarian cyst | 4 |
|  |  |  | ● ovary removal | 2 |
|  |  |  | ● ovulation | 1 |
|  |  |  | ● oxidated | 4 |
|  |  |  | ● PCOS | 22 |
|  |  |  | ● period blood mixes with cervical fluid | 9 |
|  |  |  | ● PMS | 1 |
|  |  |  | ● pregnancy | 9 |
|  |  |  | ● premenstrual bleeding | 1 |
|  |  |  | ● Qi or Blood stagnation | 1 |
|  |  |  | ● rectal bleeding | 10 |
|  |  |  | ● RLS | 1 |
|  |  |  | ● Spleen Qi Deficiency | 1 |
|  |  |  | ● Spleen Qi, Blood or Yang deficiency in TCM | 1 |
|  |  |  | ● stagnation | 1 |
|  |  |  | ● STDs | 1 |
|  |  |  | ● stress, emotional tension, or feeling stuck | 1 |
|  |  |  | ● subfertility | 1 |
|  |  |  | ● thyroid disease | 2 |
|  |  |  | ● trichomoniasis | 1 |
|  |  |  | ● uterine cancer | 3 |
|  |  |  | ● uterine fibroids | 13 |
|  |  |  | ● uterine polyps | 5 |
|  |  |  | ● vaccination | 1 |
|  |  |  | ● vaginal blocking | 3 |
|  |  |  | ● von Willebrand disease | 2 |
|  |  |  | ● Yin Deficiency with heat in TCM | 1 |
|  |  | sentiment | | 145 |
|  |  |  | ○ negative | 19 |
|  |  |  | ○ neutral | 16 |
|  |  |  | ○ positive | 110 |
|  |  | type of post | | 349 |
|  |  |  | ● advertising | 35 |
|  |  |  | ● comment | 73 |
|  |  |  | ● explaining | 47 |
|  |  |  | ● menstrual awareness | 10 |
|  |  |  | ● patient report | 18 |
|  |  |  | ● seeking help | 154 |
|  |  |  | ● spiritual | 12 |
|  | RQ1 | | | 295 |
|  |  | colour description | | 189 |
|  |  |  | ● black | 22 |
|  |  |  | ● blue | 1 |
|  |  |  | ● brick red | 1 |
|  |  |  | ● bright | 4 |
|  |  |  | ● bright red | 37 |
|  |  |  | ● brown | 71 |
|  |  |  | ● cranberry red | 1 |
|  |  |  | ● crimson | 3 |
|  |  |  | ● dark | 8 |
|  |  |  | ● dark brown | 16 |
|  |  |  | ● dark red | 15 |
|  |  |  | ● darker red/brown | 6 |
|  |  |  | ● grey | 8 |
|  |  |  | ● light brown | 3 |
|  |  |  | ● light pink spots | 1 |
|  |  |  | ● light red | 4 |
|  |  |  | ● maroon | 1 |
|  |  |  | ● orange | 6 |
|  |  |  | ● pale | 1 |
|  |  |  | ● pale red | 2 |
|  |  |  | ● pink | 25 |
|  |  |  | ● purple | 7 |
|  |  |  | ● red | 20 |
|  |  |  | ● still fresh blood | 2 |
|  |  |  | ● terracotta coloured | 2 |
|  |  |  | ● undefined | 3 |
|  |  |  | ● yellow | 1 |
|  |  | consistency description | | 123 |
|  |  |  | ● brain looking thing | 1 |
|  |  |  | ● chunky | 1 |
|  |  |  | ● coagulation | 87 |
|  |  |  | ● coffee ground inclusions | 2 |
|  |  |  | ● gloopy | 2 |
|  |  |  | ● good-quality honey | 1 |
|  |  |  | ● grainy clumps | 4 |
|  |  |  | ● light | 11 |
|  |  |  | ● mucus in MB | 1 |
|  |  |  | ● pasty | 1 |
|  |  |  | ● slimy | 2 |
|  |  |  | ● slippery | 1 |
|  |  |  | ● smooth | 1 |
|  |  |  | ● specs | 1 |
|  |  |  | ● sticky | 2 |
|  |  |  | ● stringy | 1 |
|  |  |  | ● thick | 13 |
|  |  |  | ● thin | 4 |
|  |  |  | ● tissuey flakes | 2 |
|  |  |  | ● unclear | 3 |
|  |  |  | ● watery | 8 |
|  |  |  | ● wormlike | 1 |
|  |  | other appearance description | | 71 |
|  |  |  | ○ bleeding from urethra | 1 |
|  |  |  | ○ burning period blood | 2 |
|  |  |  | ○ consistent periods | 3 |
|  |  |  | ○ irregular | 6 |
|  |  |  | ○ menstrual blood loss | 19 |
|  |  |  | ○ missing period | 1 |
|  |  |  | ○ normal | 3 |
|  |  |  | ○ not normal | 10 |
|  |  |  | ○ ovulatory spotting | 3 |
|  |  |  | ○ retrograde menstruation | 8 |
|  |  |  | ○ spotting | 16 |
|  |  |  | ○ stuck MB | 4 |
|  |  |  | ○ vaginal bleeding | 1 |
|  |  | smell description | | 23 |
|  |  |  | ● ammonia | 1 |
|  |  |  | ● applesauce | 1 |
|  |  |  | ● citrusy | 1 |
|  |  |  | ● decomposing tissue | 1 |
|  |  |  | ● foul | 1 |
|  |  |  | ● gross | 1 |
|  |  |  | ● irregular | 4 |
|  |  |  | ● metallic | 1 |
|  |  |  | ● necrosis | 1 |
|  |  |  | ● no smell | 1 |
|  |  |  | ● putrid | 1 |
|  |  |  | ● rotting | 1 |
|  |  |  | ● rotting meat | 1 |
|  |  |  | ● smells like yeast | 1 |
|  |  |  | ● sour | 1 |
|  |  |  | ● super fruity | 1 |
|  |  |  | ● sweet | 2 |
|  |  |  | ● unpleasant | 9 |
|  | RQ2 | | | 115 |
|  |  | benefits | | 29 |
|  |  |  | ○ destigmatisation | 2 |
|  |  |  | ○ early detection | 2 |
|  |  |  | ○ easily accessible | 15 |
|  |  |  | ○ fast | 4 |
|  |  |  | ○ home-based | 12 |
|  |  |  | ○ less intimidating | 1 |
|  |  |  | ○ no ethical concerns | 1 |
|  |  |  | ○ non-invasive | 10 |
|  |  |  | ○ pain-free | 3 |
|  |  |  | ○ plentiful | 1 |
|  |  |  | ○ priceworthy | 6 |
|  |  |  | ○ therapeutic use | 3 |
|  |  | company names | | 31 |
|  |  |  | ○ Diamens | 3 |
|  |  |  | ○ Endozene | 1 |
|  |  |  | ○ Fertilysis | 6 |
|  |  |  | ○ Flowintell | 1 |
|  |  |  | ○ Hello Period | 1 |
|  |  |  | ○ MensEndoDiag | 1 |
|  |  |  | ○ NextGen Jane | 1 |
|  |  |  | ○ Papcup | 1 |
|  |  |  | ○ Qvin | 15 |
|  |  |  | ○ ROSE | 3 |
|  |  |  | ○ Theblood | 1 |
|  |  | concerns | | 19 |
|  |  |  | ○ being sold off | 1 |
|  |  |  | ○ collection of MB | 1 |
|  |  |  | ○ expensive | 2 |
|  |  |  | ○ fewer stem cells than in PB | 1 |
|  |  |  | ○ loosing body autonomy | 2 |
|  |  |  | ○ menopause or no MB | 3 |
|  |  |  | ○ not getting paid | 2 |
|  |  |  | ○ political climate | 1 |
|  |  |  | ○ rather be tested by a doctor | 1 |
|  |  |  | ○ stigma | 3 |
|  |  |  | ○ toxic if consumed | 1 |
|  |  |  | ○ will take long until accessible for all women | 1 |
|  |  |  | ○ yuck factor | 1 |
|  |  | diagnostic methods | | 54 |
|  | other | | | 30 |
|  |  |  | ○ MenSCs | 17 |
|  |  |  | ○ astonished because it took so long  ○ hopes for the future | 10  3 |
|  |  |  | ○ So. A full uterus brings life. And now an empty uterus can save lives. Well. If that isn’t the most miraculous discovery since, well, ever!, I don’t know what is. | 1 |
|  |  | research | | 36 |

## Results

S3.1. General results

Table S8: Distribution of post types across social media platforms (Facebook, Instagram, Reddit, and TikTok) with the number of posts and respective percentages of total platform-specific mentions

Gr = Groundedness: number of all quotations coded by a specific code; GS=number of all posts extracted from a specific social media platform

|  | **Facebook** Gr=784 GS=92 | **Instagram** Gr=273 GS=24 | **Reddit** Gr=1194 GS=226 | **TikTok** Gr=131 GS=7 |
| --- | --- | --- | --- | --- |
| **advertising Gr=35** | 28/92 (30.4%) | 0 (0%) | 7/226 (3.1%) | 0 (0%) |
| **comment Gr=73** | 18/92 (19.6%) | 5/24 (20.8%) | 49/226 (21.7%) | 1/7 (14.3%) |
| **explaining Gr=47** | 21/92 (22.8%) | 13/24 (54.2%) | 7/226 (3.1%) | 6/7 (85.7%) |
| **menstrual awareness Gr=10** | 8/92 (8.7%) | 0 (0%) | 2/226 (0.9%) | 0 (0%) |
| **patient report Gr=18** | 1/92 (1.1%) | 0 (0%) | 17/226 (7.5%) | 0 (0%) |
| **seeking help Gr=154** | 7/92 (7.6%) | 3/24 (12.5%) | 144/226 (63.7%) | 0 (0%) |
| **spiritual Gr=12** | 9/92 (9.8%) | 3/24 (12.5%) | 0 (0%/0%) | 0 (0%) |
| **Totals** | 92 | 24 | 226 | 7 |
